# Medical students and metrics: seven techniques for a win-win situation

**DOI:** 10.1186/s12245-019-0230-2

**Published:** 2019-05-06

**Authors:** Tracy MacIntosh, David C. Lebowitz, Latha Ganti

**Affiliations:** Emergency Medicine, University of Central Florida, Orlando, FL USA

**Keywords:** Medical students, Physician productivity, Teaching physician

## Abstract

**Background:**

The authors present seven winning strategies for maintaining a rich academic environment for learners while working in a busy emergency department with expected productivity metrics.

**Methods:**

This is a descriptive paper based on existing literature and on the authors’ experience.

**Results:**

Winning strategies to improve ED throughput while also supporting the mission of medical education and improving the learning environment for students include the following: (1) attending first, (2) provider in triage, (3) mobile workstations, (4) patient education, (5) bedside patient presentations, (6) dedicated teaching resident, and (7) thoughtful scheduling.

**Conclusions:**

The authors present seven practical strategies that are portable to many settings.

## Background

With the expansion of medical schools and emergency medicine residency programs, an increasing number of community emergency departments (EDs) are developing educational programs. Additionally, established emergency training programs must continue to maintain their strong educational missions while focusing on improving their internal metrics. In both of these types of training environments, educators and administrators have priorities that may seem at odds. Bhat et al. examined productivity of attending physicians working paired with medical students compared to those working alone and found that overall productivity was the same [1]; however, examining specific ED workflow measures may reveal additional challenges and opportunities. The Center for Medicare and Medicaid Services (CMS) established a number of emergency department throughput measures as part of the clinical quality measures [2, 3]. While the majority of measures are independent of the presence of learners, ED length of stay (LOS) for admitted and discharged patients may be directly impacted by the additional time needed to educate and supervise students and residents in the ED, which may also impact the number of patients who left without being seen (LWBS) due to increased waiting room times. LOS is therefore the primary focus of interventions and strategies reviewed in this article.

### What is the impact of medical students and residents on emergency department length of stay?

A number of studies have evaluated the impact of medical students and residents on ED LOS. The studies vary in terms of their methodology, with most comparing overall ED LOS, rather than looking at the individual patient as the level of analysis.

Gerbeaux et al. took advantage of a 4-day medical student strike in France to compare ED LOS, matching by days of the week for the week of the strike compared to the same days of the week prior. The authors found that the presence of medical students was associated with a 31-min longer LOS (95% CI 24–38 min) [4]. Using similar methods, Ioannides et al. found that student presence was associated with a 5-min increase in overall ED LOS comparing the 3-week presence of students in the ED to the week where the same students rotate in the operating room for anesthesiology instead [5]. Important limitation of both of these studies is that they do not identify what proportion of patients was seen by students or the role of students.

Using US National ED data, a number of studies have supported the association between teaching hospitals and increased ED LOS. Looking at a total of 424 hospitals, Pines et al. found that teaching hospitals were associated with 8-min longer wait times, 20-min longer discharge LOS, and 36-min longer admitted LOS (*p* < 0.05) [6].

The most methodologically robust study evaluated individual patient encounters and analyzed them by provider. Delaney et al. found that patients seen with students were associated with a 24-min longer discharge LOS, compared to patients seen only by attending physicians (*p* < 0.001), but had a 2-min shorter door-to-medical provider time (*p* < 0.001) [7].

Overall, the data demonstrate strong evidence that the presence of medical students is associated with longer ED LOS. Given that the CMS has identified ED discharge LOS as a clinical quality measure [8], it is important that EDs are able to strengthen their educational goals and programs without compromising on ED throughput and metrics. We have identified and implemented seven techniques at our ED that have been associated with successful improvements in our discharge LOS times as we have served as a core clinical site for the University of Central Florida with growing numbers of both home and away student rotators.

## Methods

This is a descriptive paper describing both existing evidence-based practices from current literature and also based on the authors’ experiences and techniques teaching medical students in a busy emergency department environment. The authors practice in a community hospital that sees over 80,000 emergency department visits. The institution is the primary teaching hospital for the local medical school and home to residency training programs in emergency medicine, surgery, internal medicine, psychiatry, obstetrics and gynecology, and a transitional year program. The medical school sends students to rotate in the emergency department during their fourth (final) year of training.

**Fig. 1:**
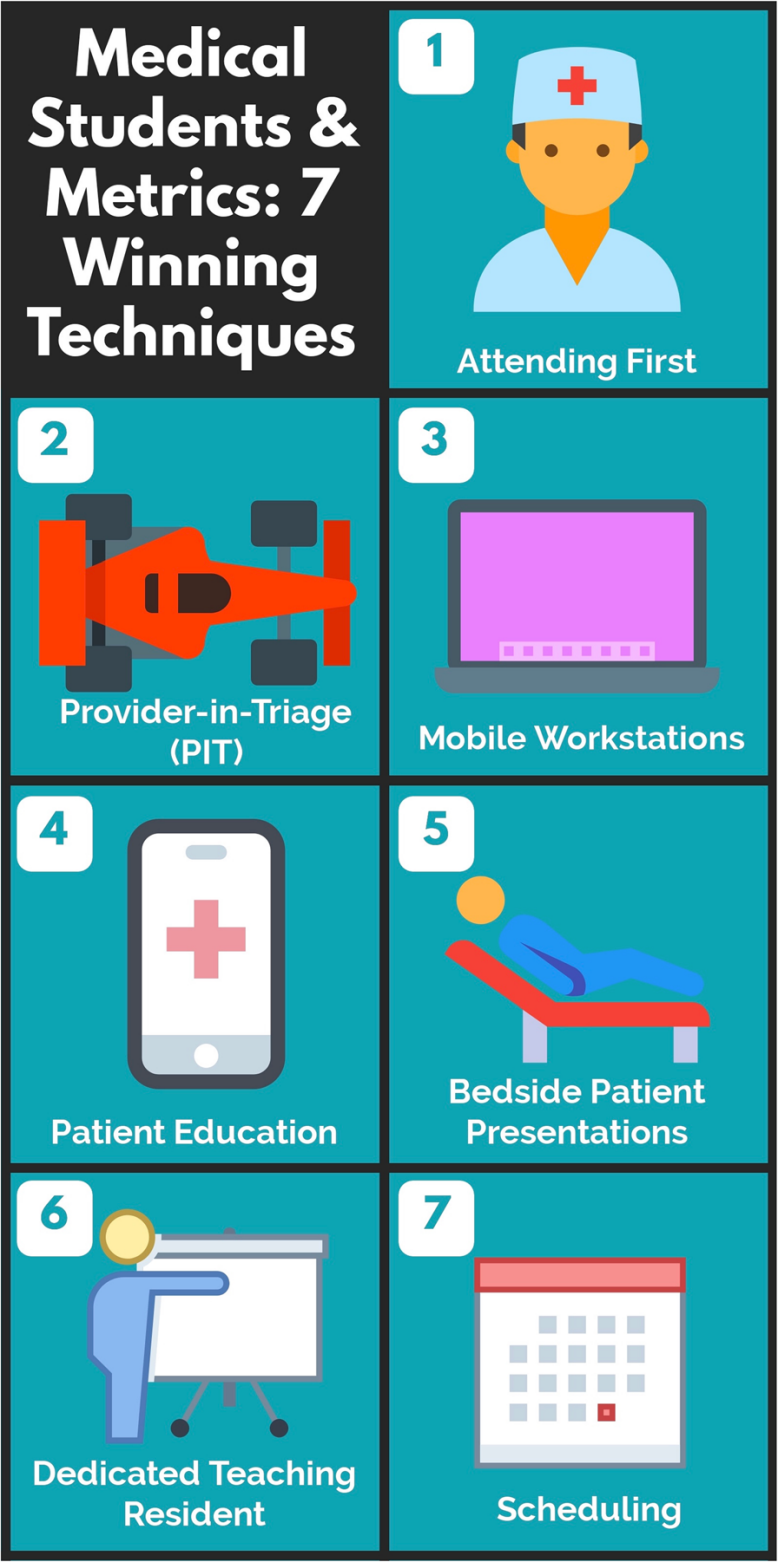
Winning strategies for improving ED throughput while also supporting the mission of medical education

This article reviews the literature on the impact of medical students and residents on the most important ED metrics and provides seven practical techniques to improve ED throughput while also supporting the mission of medical education and improving the learning environment for students (Fig. 1).

## Results

### Attending first

Initiating the work-up as soon as possible is an important strategy to promote ED efficiency, decrease LOS, and improve patient satisfaction. The attending physician should be first to greet and then screen the patient and perform a brief history and pertinent physical exam. While many teaching hospitals are overt in explaining the role of learners in their care, many patients may still not understand the direct impact of medical students on their care [9] and this initial greet provides an opportunity to address any potential concerns about the teaching process and role of the student. The preceptor can obtain permission from the patient to be fully evaluated by a student, who can then be formally introduced to subsequently complete their comprehensive history and physical exam. During this time, the attending proceeds to input orders and initiate the work-up in parallel in the background. When the attending greets the patient before the student, patients can gain a greater appreciation for the role of the student as part of the care team, and the attending can expedite time to diagnostic test orders and results.

### Provider in triage

Enlisting an advanced practice provider (APP) or physician in triage (PIT) will significantly reduce the door-to-medical-provider time (DTMP) for walk-in patients and can also facilitate the rapid initiation of the patient’s ED evaluation and management. Partovi et al. [10] have demonstrated that faculty triage for walk-in patients had an 18% reduction in LOS and 46% reduction in LWBS rate compared to those without physician triage. The disadvantage of PIT and having the attending see the patient first are that the orders are already being placed in the electronic health record, and students may access to orders and/or results prior to independently evaluating the patient. This can cause bias due to premature closure and anchoring and may compromise the development of their critical thinking. Therefore, to decrease the influence of previous orders, we recommend that students be temporarily blinded to the EHR if they are seeing a patient that already has orders placed.

### Mobile workstations

The increased emergency department volumes and heavy reliance of computers for charting, record review, order entry, and results review can adversely impact time spent in direct patient care [11]. At the same time, it remains essential for attendings to perform direct observations of students at the bedside to hone and correct history and physical exam skills. One technique to resolve these needs is through a mobile computer or tablet that the preceptor can utilize. This is a true win-win as it allows physicians to directly observe the student perform a history and physical exam while simultaneously placing orders in the EHR or reviewing medical records and allows for a quick initiation of the work-up and data acquisition combined with bedside teaching. Physicians may choose to give direct feedback to the student in real-time at the bedside or in a private setting following the patient evaluation.

### Patient education

Another strategy that uses mobile computing is through the use of medical education applications (apps), such as anatomy apps and available medical illustration apps (e.g., DrawMD [12]) that learners can use to educate patients on their disease process. These apps are available on most devices and phones and can increase patients’ comprehension of their condition and may lead to improved compliance, potentially decreasing bounce backs. Limited evidence also suggests that these types of apps may improve patient satisfaction [13]. Furthermore, physicians may choose to observe these discharge discussions, allowing them to more fully evaluate students’ clinical knowledge and communication skills.

### Bedside patient presentations

Time dedicated to bedside patient presentations is on the decline, and though learners report that it may be more challenging for them, they do appreciate its educational value [14], and bedside presentations may even be preferred by patients themselves [15]. Bedside presentations may reduce duplication that can result after a separate and geographically isolated presentation, followed by the physician repeating relevant questions and physical exam maneuvers. Specifically, the student presents the history and physical examination as well as their differential diagnosis and plan to the attending at the bedside of the patient. This leads to increased patient involvement and patient-centered care. Often, the patient may fill in gaps in the student’s presentation, leading to a more accurate portrayal of the patient.

### Dedicated teaching resident

Instituting a teaching resident is another strategy that can indirectly help with hospital metrics and significantly augment medical student teaching. This removes the teaching burden from the attending physician or clinical resident and enables the teaching resident to focus only on teaching the medical student. Having a dedicated teaching resident can be challenging in a resource-limited ED. One way to combat this is by creating a medical education elective with the required teaching shifts.

### Scheduling

Thoughtful scheduling can also help in achieving metric goals. A lean or fast track in the emergency department is often fast-paced with a high turnover of patients. Having a student there can lead to potential delays and can slow down the lean process. Depending on the student load, it is encouraged to decrease the scheduling of students in this type of emergency department setting and schedule the students more heavily in higher acuity areas. It is important to note that there is a value in students seeing low acuity patients, but in order to decrease delays and increased length of stays, having a lighter student load may help. Higher acuity patients use more resources which contribute to the bulk of the length of stay. Therefore, acute patients represent an ideal area for teaching because they afford more time for medical student evaluation. Delaney et al. found that laboratory studies added an average of 150 min to the LOS and radiology adding 108 min [7]. These delays waiting for results can be seen as an opportunity for teaching and student re-evaluations of patients.

## Discussion

Faculty and ED leaders at teaching institutions can be purposeful in guiding the department’s educational strategies for medical students in order to optimize teaching while optimizing ED length of stay and other important metrics. This article reviews the existing literature on the impact of medical students on ED length of stay and finds that there is a lack of consistent evidence to suggest that their presence adversely impacts these metrics. We provide seven practical techniques to improve ED throughput while increasing learning opportunities for medical and other students. In addition to clinical teaching, educators may also consider including students in administrative educational opportunities. For example, there is evidence that students can be effective team members of ED quality improvement (QI) projects. In a study of QI initiatives, Manning et al. found that with appropriate leadership, third-year medical students could effectively learn about and become effective members of QI teams [16].

The presence of medical students may also have a beneficial effect on patient satisfaction in the ED. Using a before-and-after design with the introduction of medical students into two community-based hospitals, Kiefer et al. found that the presence of students did not adversely impact patient satisfaction and may have had a positive impact on the overall perception of the ED [17]. It is important that as teaching programs bring medical students into the fold, patients are appropriately educated on the role of medical students on the healthcare team in order to ensure that students do not adversely impact the patient-physician relationship [9].

The ED is an ideal clinical environment with high clinical acuity, undifferentiated patients, and the full gamut of medical specialties. More medical schools are appreciating the merits of an ED rotation, and a growing number of hospitals are hosting students for required and elective rotations. We have outlined seven practical strategies that ED administrators and attendings can implement to strengthen the educational experience of students without increasing length of stay or adversely impacting other ED metrics including LWBS and patient satisfaction.

## Conclusion

Seven winning strategies for improving ED throughput while also supporting the mission of medical education include the following: (1) attending first, (2) provider in triage, (3) mobile workstations, (4) patient education, (5) bedside patient presentations, (6) dedicated teaching resident, and (7) thoughtful scheduling. These practical strategies are transferrable to many settings.

## Abbreviations

APP: Advanced practice provider
CMS: Centers for Medicare and Medicaid Services
ED: Emergency department
LOS: Length of stay
LWBS: Left without being seen
PIT: Physician in triage
